# Description of an Innovative Pediatric Individualized Therapeutics Clinic: Working toward Precision Drug Therapy

**DOI:** 10.3390/children6020035

**Published:** 2019-02-25

**Authors:** Tracy L. Sandritter, Jean C. Dinh, Jennifer A. Wagner, Jennifer A. Lowry

**Affiliations:** Division of Clinical Pharmacology/Medical Toxicology and Therapeutic Innovation, Children’s Mercy Hospital, 2401 Gillham Road, Kansas City, MO 64108, USA; jdinh@cmh.edu (J.C.D.); jawagner@cmh.edu (J.A.W.); jlowry@cmh.edu (J.A.L.)

**Keywords:** pharmacogenetics, personalized medicine, pediatrics, multidisciplinary clinic, adverse drug reaction

## Abstract

The GOLDILOKs^®^ (Genomic and Ontogeny-Linked Dose Individualization and cLinical Optimization for KidS) Clinic aims to provide families and physicians with data to make more informed decisions with regard to pharmacological therapy by using innovative therapy and genomic technologies. The objectives are two-fold: (1) To describe the utility of the GOLDILOKs^®^ Clinic to referring prescribers by evaluating the type of referrals made to the GOLDILOKs^®^ Clinic and (2) to assess the most often utilized technologies (e.g., genotyping) completed to formulate therapy recommendations. Patient data from July 2010 to June 2016 was retrospectively reviewed following Institutional Review Board (IRB) approval. The GOLDILOKs^®^ Clinic evaluated 306 patients and had increases in annual referrals from 14 in 2010–2011 to 84 in 2016–2017. The children that were referred were predominately Caucasian (82%) and male (59%) with an average age of 12.4 ± 5.9 years. Subspecialty versus primary care referrals accounted for 82% and 18% of referrals, respectively. Adverse drug reactions (n = 166) and poor medication response (n = 179) were the major reasons for referral. However, it must be noted that patients could have multiple reasons for referral. Pharmacogenetic results were extensively used to provide guidance for future therapy in patients with medication-related problems. Genotyping of drug metabolizing enzymes and drug target receptors was performed in 221 patients (72.2%). Recommendations were fully accepted by 63% and partially accepted by 22% of internal provider referrals.

## 1. Introduction

Precision medicine is a concept that incorporates high throughput data to characterize diagnosis and optimize therapeutics, with the intent to improve patient health outcomes [1,2]. With this in mind, the goal of a precision medicine clinic from the therapeutic perspective of patient care is to determine the right medication and dose for each individual patient instead of utilizing a “one size fits all” approach. A common approach of precision medicine clinics has been to focus on pharmacogenetic information to put an end to the trial and error process of finding the best medication. However, personalizing medicine for a patient must also incorporate knowledge of the pharmacologic and pharmacokinetic (PK) properties of medications. The medication–response relationship is multifactorial, including compliance, absorption, distribution, receptor interaction, metabolism, elimination, and a clear diagnosis with appropriate therapy. All these variables need to be considered when providing precision medicine. Understanding what might be the perfect medication for a child is not as simple as ordering the pharmacogenetic tests associated with medication metabolism. Genes code for both PK (e.g., drug metabolizing enzymes) and pharmacodynamic (PD) targets (e.g., drug transporters or target receptors). Genetic variations can lead to differences in drug efficacy or the potential for adverse reactions in different patients. Choice of therapy for a particular patient therefore has to take into consideration multiple potential variations related to different combinations of these variations. Likewise, understanding the subtleties of different drug formulations can improve the choice of alternative products that may optimize clinical effects, while minimizing side effects.

Several technologies are available to aid in individualizing medicine, including single nucleotide polymorphism genotyping, estimation of genotype via a phenotype study, and measurement of drug concentrations in the body to understand drug disposition in a particular patient. Ideally, pharmacogenetic testing would be performed preemptively, as opposed to reactionary testing to guide future therapies. However, reactionary testing may be more prominent due to the challenges of widely implementing pharmacogenetic testing and the relatively new availability of this testing in the outpatient clinical setting [3,4,5]. There are examples in pediatric cardiology, pediatric oncology, as well as psychiatry, where pharmacogenetic information can be utilized to personalize patient care [6,7,8,9,10]. This brief report will provide an overview of the GOLDILOKs^®^ (Genomic and Ontogeny-Linked Dose Individualization and cLinical Optimization for KidS) Clinic at Children’s Mercy Hospital, which was established in 2010 as part of our initiative to integrate the advancements of genomic technologies and pharmacogenetic research directly into pediatric patient care. Our approach is to consider additional factors which may impact the medication–response relationship and to incorporate PK, PD, and pharmacogenetic information when providing medication therapy recommendations.

## 2. Materials and Methods

A retrospective review of the GOLDILOKs^®^ Clinic database at Children’s Mercy Hospital (CMH) was conducted following approval from the Children’s Mercy Institutional Review Board (IRB) (ID#15050182, approved 27 September 2015). All patients referred to the GOLDILOKs^®^ Clinic are entered into our clinic database and are seen without exclusion. Patients who do not show for the appointment are still included in the database with the information available to us at the time of initial referral. Demographic information for all patients in the GOLDILOKs^®^ Clinic database between 1 July 2010 and 30 June 2016 were included in this analysis. Patients who did not show for the clinic visit were counted in the total number of patients scheduled. Patients seen after 30 June 2016 were excluded from this description of the GOLDILOKs^®^ Clinic.

**Figure 1:**
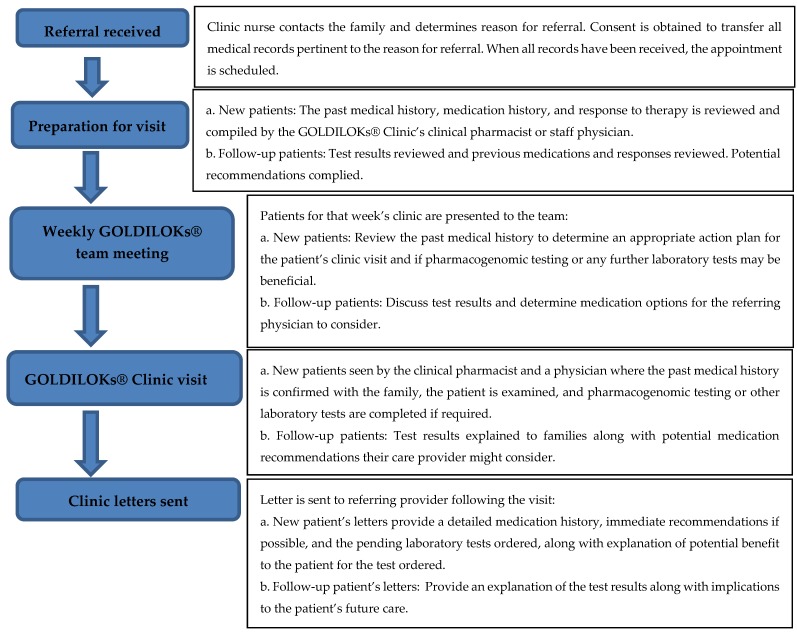
GOLDILOKs^®^ (Genomic and Ontogeny-Linked Dose Individualization and cLinical Optimization for KidS) Clinic flow chart.

GOLDILOKs^®^ Clinic Operations (Figure 1): Patients are referred to the GOLDILOKs^®^ Clinic via multiple processes, which include internal and external healthcare providers in both medical subspecialties and primary care settings, as well as self-referrals by the patient’s legal guardian. Upon receipt of the referral, the clinic’s nurse coordinator contacts the family to determine the reason for referral and to obtain consent from the legal guardian to transfer all medical records pertinent to the reason for referral, including prior medications and the corresponding response to therapy. Once all the pertinent medical history is received for review, the family is contacted to schedule the initial clinic visit. The past medical history, medication history, and response to therapy is then reviewed and compiled by the GOLDILOKs^®^ Clinic’s clinical pharmacist or staff physicians. This information is presented at the weekly multidisciplinary GOLDILOKs^®^ Clinic’s team meeting. The multidisciplinary team includes nurses, pharmacists, physicians, clinical pharmacologists, clinical pharmacology fellows, and research scientists (Ph.D.). Together the team reviews the patient’s past medical history to determine an appropriate action plan for the patient’s clinic visit and if pharmacogenomic testing or any further laboratory tests may be beneficial. Patients are then seen in the clinic by the clinical pharmacist and a physician, where the past medical history is confirmed with the family, the patient is examined, and pharmacogenomic testing or other laboratory tests are completed if required. Patient information is collected and stored in the GOLDILOKs^®^ Clinic database which is maintained in a Research Electronic Data Capture (REDcap) database (Vanderbilt University, Nashville, TN, USA). REDcap is a password protected database used for our record keeping and tracking of our clinic patients. After the visit is completed, a letter with possible immediate therapy recommendations and pending laboratory results is sent to the referring physician and primary care provider. If applicable, once the results are available for review, the patient is scheduled for a follow-up visit to discuss the meaning of the results and any additional therapy recommendations that might be appropriate for the patient’s care. Thus, patients are typically seen in our clinic twice. The clinic serves in an advisory or consultative capacity to the patient’s prescribing physicians, with specific emphasis on the medication options considering the PK, PD, and pharmacogenetics that might be available.

Clinical and laboratory data included in this analysis were extracted from the GOLDILOKs^®^ Clinic database, which is maintained in REDcap. Data collection included basic demographic data; underlying diagnoses; reason for referral to the clinic; referring physician information; medications that the patient is on at the time of the visit; prior medications for which there were adverse reactions or poor response to treatment; pharmacogenetic testing and other laboratory, personalized medicine, or diagnostic tests ordered; and recommendations made to the referring physician. If the patient was referred by a CMH provider, a chart review was conducted within 6 months of the visit to determine if there was acceptance, partial acceptance, or non-acceptance of the recommendations by the referring provider. Acceptance was defined as all recommendations made were accepted. Partial acceptance was defined as some of the recommendations were accepted and non-acceptance was defined as none of the recommendations were accepted.

Descriptive statistics were used to describe the referrals made to the GOLDILOKs^®^ Clinic and to describe the laboratory evaluation or testing completed to help provide recommendations for altering therapy to the referring health care provider.

## 3. Results

Since the start of the GOLDILOKs^®^ Clinic in July 2010, there were 315 referrals placed (initial clinic visit), 164 follow-up visits, and 5 patients who were seen for a third time. Taking into account duplicate patients and rescheduling, the total number of individuals actually seen between 1 July 2010 and 30 June 2016 was 306. The number of patients seen during each year of clinic operation is presented in Table 1. The patients seen were predominately male (59%). Self-reported race included Caucasian (n = 254, 81.9%), multiracial (n = 18, 5.8%), African American (n = 15, 4.8%), Asian (n = 12, 3.8%), Hispanic (n = 7, 2.3%), Middle Eastern (n = 2, 0.6%), and other (n = 2, 0.6%). The average age was 12.4 ± 5.9 years. Fifty-three patients were adopted from the United States (n = 41), China (n = 8), Korea (n = 1), and unknown countries (n = 3). Fourteen patients were initially seen as an inpatient consult with follow-up appointments in clinic.

Referrals to the GOLDILOKs^®^ Clinic were predominately made by pediatric medical subspecialists compared to pediatric primary care providers (n = 257 (82%) vs. n = 57 (18%), respectively). One patient was noted to be a self-referral. Additionally, the majority of referrals were made by care providers within the host institution. Among the subspecialty of care providers, psychiatry represented the majority of referrals at 73% (Table 2). Referrals included evaluation of multiple adverse drug reactions (166/401, 41%), poor therapeutic response/tachyphylaxis to prescribed medications (180/401, 45%), genotyping requested by the physician or the family (24/401, 6%), interpretation of genotyping previously obtained (14/401, 3.5%), concern for potential QT interval prolongation due to a need for anxiolytics or antidepressants (3/401, 0.7%), concern for potential drug–drug interactions given need for many medications (2/401, 0.5%), previously seen and genotyped but continues to struggle (2/401, 0.5%), concern for therapy initiation based on family history of adverse reactions (1/401, 0.2%), and other reasons (9/401, 2%) (Table 3). Some patients had more than one reason for referral (n = 106). The most common underlying diagnoses included Attention Deficit Hyperactivity Disorders (n = 164), anxiety disorders (n = 132), Autism Spectrum Disorders/Pervasive Developmental Disorder (n = 84), gastrointestinal illnesses (n = 70), tic disorders (n = 50), depression (n = 46), seizures (n = 45), sleep disorders (n = 26), and syndromes/chromosomal anomalies (n = 35).

**Figure 2:**
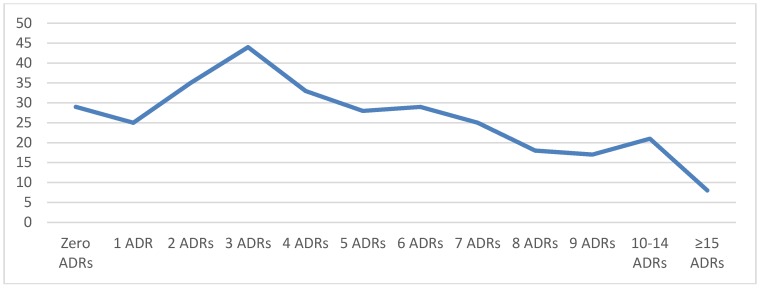
Number of patients with adverse drug reactions (ADRs).

The mean number of medications taken per patient upon initial evaluation in the clinic was 5.2 ± 3.8 (median: 5, mode: 4, range: 0–25). The top 15 medications were Melatonin (n = 74), guanfacine (n = 68), cetirizine (n = 45), methylphenidates (n = 42), fluticasone (n = 41), amphetamine salts (n = 39), clonidine (n = 39), polyethylene glycol 3350 (n = 39), montelukast (n = 34), albuterol (n =3 3), fluoxetine (n = 33), sertraline (n = 30), aripiprazole (n = 26), dexmethylphenidate (n = 23), and trazodone (n = 22). The most commonly used antiepileptics were topiramate, valproic acid, and levetiracetam. Twenty-three patients were on no medications at the time of the first clinic appointment. The mean number of adverse reactions to medications per patient at the first clinic visit was 5 ± 4.1 (median: 4, mode: 3). The number of prior medication adverse reactions at the time of the first visit was variable, ranging from 0 to 34 (Figure 2).

**Figure 3:**
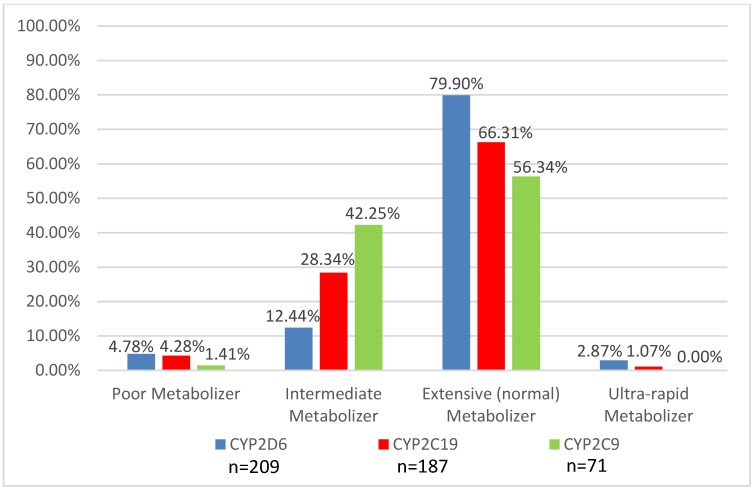
Distribution of predicted phenotypes for CYP2D6, CYP2C19, and CYP2C9 in a GOLDILOKs^®^ Clinic.

**Figure 4:**
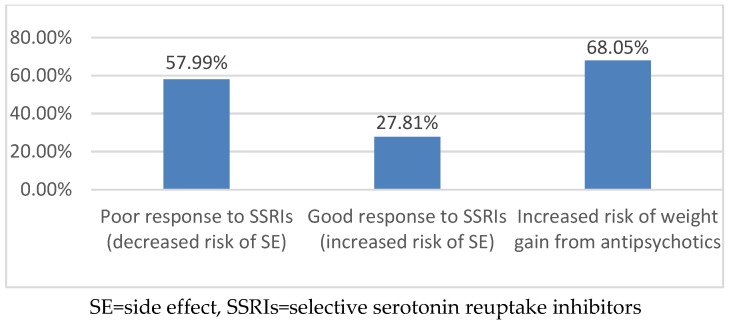
Serotonin 2A/2C receptor phenotype predictions in a GOLDILOKs^®^ Clinic.

Genotyping was obtained to assist in medication recommendations in 221 patients (72.2%). Cytochrome P450 2D6 (*CYP2D6*) genotyping was the most common gene tested (99%), followed by Cytochrome P450 2C19 (*CYP2C19*) (88%) and the serotonin 2A and 2C receptors (*HTR2C* and *HTR2A*) (76%). Other genotyping tests performed included *CYP2C9* (14%); *CYP3A5* (1%); *SLC6A4* (25%); the gene that encodes for the serotonin reuptake transporter (18%); *DRD3*, the gene that encodes the dopamine 3 receptor (14%); and *DRD4*, the gene that encodes for the dopamine 4 receptor (12%). Figure 3 and Figure 4 provide the phenotype predictions, based solely on the genotype results, for the CYP enzymes most commonly tested in the GOLDILOKs^®^ Clinic and the serotonin 2A/2C receptor phenotype predictions in our population.

Genotyping assessments were not the only laboratory tests ordered. Other laboratory tests obtained included: Complete blood count (n = 32), iron studies (n = 26), basic metabolic panel (n = 15), liver function test (n = 17), thyroid studies (n = 11), therapeutic drug monitoring (n = 6, oxcarbazepine × 1, lithium × 2, valproic acid × 1, clozapine × 1, clonidine × 1), area under the curve assessment × 2 (phenobarbital, valproic acid), and phenotype evaluation (n = 2) which predicted cytochrome P450 2D6 function by using dextromethorphan (1 = normal metabolizer, 1 = indeterminate). Forty-nine other miscellaneous tests were ordered.

For patients whose prescribing doctors were internal to CMH, chart review found that 63% of recommendations made to the referring physician were accepted, with an additional 22% of recommendations partially accepted. Recommendations were not accepted in 14% of the patients referred to the clinic. Lastly, for 1% of the patients, data were not obtained due to lost to follow-up when the patient switched to a provider outside of CMH.

## 4. Discussion

To our knowledge, this is the first report describing an outpatient pediatric precision medicine clinic in which the entire medication–exposure relationship is assessed. Since 2010, we have successfully implemented a novel multidisciplinary pediatric personalized medicine clinic in which the patients have access to recommendations and education from pediatricians, pharmacists, clinical pharmacologists, researchers, and those in other disciplines working together to provide medication therapy recommendations to referring providers to address this need. Our clinic has expanded from 14 referrals during the first year of operation to 84 referrals during the sixth year of operation. Multiple variables are considered including PK, PD, and pharmacogenetic testing when appropriate. The majority of referrals to the GOLDILOKs^®^ Clinic were to advise in scenarios where patients had experienced multiple adverse drug reactions (ADRs), poor therapeutic response, or tachyphylaxis to prescribed medications. Referring care providers and parents appear particularly interested in implementing pharmacogenetic testing to assist in developing treatment plans for children. However, pharmacogenetic testing is not the only resource a personalized/precision medicine clinic might use.

The most common reasons for referral to our clinic were history of multiple adverse drug reactions and poor therapeutic response/tachyphylaxis to prescribed medications. To investigate the cause(s) of ADR or treatment failure, we conducted a thorough chart review and considered factors such as compliance, absorption, distribution, drug-receptor interaction, metabolism, elimination, and diagnosis. In addition, a patient and family interview is completed to understand the presentation and timeline of the ADR. A thorough medication history is obtained, including a complete list of prescription and over the counter medications that the patient is or was taking, given that the likelihood of developing an adverse interaction increases with the number of drugs prescribed [11,12]. For instance, data suggest that if five drugs are given simultaneously, the chance of an adverse interaction occurring is approximately 50% [13]. The patients referred to this clinic were typically on several medications (mean (SD) = 5.2 (3.8)) with a history of multiple prior adverse reactions (mean (SD) = 5 (4.1)). As such, our clinic population is at high risk for morbidity from ADRs based on the polypharmacy seen.

ADRs are of two types: Pharmacologic, dose-related (Type-A) and idiosyncratic, non-dose related (Type-B). Type-A ADRs, the most common reactions, are dependent on the dose and reversible with dose reduction [14]. As such, pharmacogenetic testing might help in the correlation of ADRs with a medication. Besides dose, there are multiple other factors that can contribute to Type-A ADRs, including formulation variations, PK variation, and drug–drug interactions, which we also take into account during each of our clinic visits with a patient. Type-B reactions may be impacted by the body’s drug metabolizing system due to potential accumulation of toxic metabolites [15] which may contribute to ADRs. Additionally, Type-B reactions are also thought to be due to the immune system, as not all Type-B reactions can be simply explained by alterations in metabolism. Pharmacogenetic testing for variations in drug metabolism can help explain ADRs with an estimated 15%–30% of variable drug response due to genetic polymorphisms [16,17]; however, it is important to note that the majority of polymorphisms are rare with minor allele frequencies (<1%) [18]. In our clinic, this is evident based on the limited number of poor and ultra-rapid metabolizers (Figure 3), despite the high number of ADRs (mean 5 ± 4.1). Therefore, while pharmacogenetic variability may explain some of the ADRs observed in our population, it would appear that, for the majority of patients, their reason for drug response/non-response is likely multi-factorial and requires multidisciplinary collaboration to interpret and guide therapy. PD (drug-receptor interactions) variability also needs to be considered when assessing medication response (Figure 4); unfortunately, current data related to the PD response is not as extensive as available PK data. In addition to understanding the pharmacogenetics factors contributing to ADRs in our clinic population, this multidisciplinary clinic serves to address all possible contributing factors to ADRs.

Genotyping is often requested not only to understand why the child has frequent ADRs to medications, but also to choose initial therapies, or to re-challenge the child with medication that will be less likely to result in a repeat ADR. In our clinic, pharmacogenetic testing was stated at a reason for referral ~6% of the time. Twenty-four (8%) family requests for genotyping were also noted. Consistent with available literature, parents are concerned that medications have significant side effects [19]. There is concern that giving medications to treat behavior problems in pediatric patients will have a long-term negative impact on development. Additionally, medications can impact mood and personality, with parents often describing a flat, “zombie”-like effect which may decrease the utilization of medications or delay treatment of the underlying behavior problem [20]. Based on a survey of public attitudes [21], pharmacogenetics testing is desirable for a variety of reasons, including prediction of drug effectiveness, initial dose determination, and concerns related to adverse drug reactions. In this survey, participants were interested in pharmacogenetic testing to predict mild (73 ± 3.29%) or serious (85 ± 2.91%) side effects, to use this information to guide dosing (91%), or to assist with drug selection (92%). In our clinic, we suspect that pharmacogenetic testing with evaluation by an integrative care team was sought out by providers and families based on previous experience with medication options and to support future plans for care. Thus, parents and prescribers seek out additional information such as pharmacogenetic testing that might provide insight into the next steps for medication therapy.

Anecdotally, parents express concerns about a trial-and-error approach for determining the correct medication for their child and, if given the option, would rather make more informed decisions for their child’s care through pharmacogenetic testing. Of interest, 23 referred patients were on no medications at the time of their initial visit but were considering medications in the future. Additionally, the percentage of adopted children who presented to the clinic (53/306, 17.3%) is substantially higher than the national average of 7% according to the 2010 US Census [22]. Adoptive parents are unlikely to have knowledge of prior family history of their adopted child, knowledge which could have assisted providers with choosing initial medication management. For these families, pharmacogenetic testing might offer some confidence in the choice of medication. This is speculative and requires further investigation, especially to determine if the patient benefited from less medication adjustments or avoided side effects due to preemptive pharmacogenetic testing.

Pharmacogenetic testing is frequently sent to commercial laboratories, which then provides the pharmacogenetic results and some generic medication recommendations. However, determination of the optimal medication for a patient is not as straightforward as would be anticipated, given the various combinations of PK and PD related genes that need to be considered for each individual medication. Another difficulty in utilizing the information is that the phenotype prediction provided on the reports is based solely on the patient’s genotype and does not account for drug–drug interactions, drug–food interactions, underlying illness, or ontogeny in the pediatric population, which can also impact the patient’s phenotype. Additionally, for some drug–gene interaction pairs, guidelines such as those available on www.pharmgkb.org or dosing information with the drug’s package insert may not be available. Primary care providers are often not trained on the use of these panels, leaving providers with additional questions but no easy access to answers. Thus, specialty precision medicine clinics such as ours can help prescribers understand the reports more specifically to an individual patient.

As noted above, providers and families have sought out pharmacogenetic testing as a means to determine the optimal medication for the patient. However, pharmacogenetic testing is not the only tool utilized to provide precision therapeutics for children in our clinic because for many medications genotyping predicting response (PD) to the medication may not be available. Thus, our clinic also takes into account other variables that might impact response to treatment such as compliance, drug formulations, and medication history. For medications to be effective, patient compliance has to be considered. Parental apprehensions regarding the necessity of medication impacted compliance such that parents who expressed higher levels of skepticism towards a medication were more likely to have poor adherence (*p* < 0.05) [23]. Conceivably, pharmacogenetic information may ease apprehension toward future medications and possibly impact compliance, although this needs to be studied. In some situations, PK information related to medication dosage forms may be more valuable compared to pharmacogenetic results. For example, while there are companies offering pharmacogenetic Attention Deficit Hyperactivity Disorder (ADHD) panels, results gained from this testing do not provide providers with specific recommendations outlining which type of stimulant might be better for the patient (amphetamine versus methylphenidate), because currently there are no pharmacogenetic studies comparing the efficacy of different stimulants to each other. Current evidence related to prediction of methylphenidate efficacy is predominately from non-randomized association studies in which genotypes are sorted by response and non-response [24,25]. Multiple reviews comparing the pharmacokinetics of the available stimulant formulations have been published and are utilized in our clinic to help provide recommendations related to the stimulant medications [26,27]. Use of this information can help tailor the ADHD medication regimens for a patient by selection between the various short-acting and long-acting formulations and assessing of the peak time of day with poor symptom control. Another benefit of our clinic is that we provide the primary prescriber with an extensive pharmacologic review and evaluation of previous medication exposures and responses from all the patients’ multiple providers, past and present, to the extent that the records are made available to us. Insight provided by such an extensive medication history from outside reviewers may help providers consider medications for re-challenges and other options. Finally, multidisciplinary precision medicine clinics such as ours can help prescribers with other medication related dilemmas and interesting therapeutic challenges. For example, our clinic has assisted with subcutaneous octreotide and intranasal oxytocin for a patient with panhypopituitarism and intravenous iron infusions for patients with restless leg syndrome and insomnia who were unable to tolerate multiple different enteral iron preparations or lack of success replacing ferritin with confirmed enteral compliance.

As our clinic is a consultative service clinic to long-term care providers, the ability to directly assess the outcomes of all our recommendations is difficult, as we do not have access to patient outcomes long-term. This is a retrospective, descriptive study of the implementation of a GOLDILOKs^®^ clinic and assists with understanding the reasons for which providers and families request genotyping. Thus, moving forward, we intend to use this information to effectively choose outcome parameters that might be implemented to determine the benefits to the patient and the cost effectiveness of clinics such as ours. However, for referring prescribers within our health care system, we were able to determine that about 63% of recommendations made were accepted, with an additional 22% of recommendations partly accepted. Partly accepted means that some of the recommendations made were accepted. This is likely because we provide multiple options simultaneously for consideration to the referring physician who follows the patient long-term. Given the multiple options that might be appropriate to a patient’s care and because frequently multiple medication changes are not made simultaneously, in order to better access the clinical impact of the medication change in a particular patient, we would anticipate that not all the recommendations would be completely accepted at the time the chart was reviewed.

## 5. Conclusions

Pediatric personalized medicine encompasses additional challenges as children undergo dynamic growth and development and have demonstrated different responses to drugs. A multidisciplinary pediatric precision medicine which can consider PK, PD, and pharmacogenetic factors can help providers and families find treatment options for children who have experienced poor medication response and adverse reactions. Since the medication–response relationship is multifactorial, it will be important to further assess the benefits of a precision medicine clinic, including newer technologies such as pharmacogenetic testing. The pediatric personalized medicine clinic model is likely generalizable and applicable at academic hospitals to select patients in need of additional resources. Our paper shows there is a need and demand for evaluation and follow-up within a formal clinic setting. Continued research is needed to determine whether these findings translate to improved outcomes, streamlined medication selection, fewer adverse drug reactions, patient satisfaction, and provider satisfaction.

## Figures and Tables

**Table 1:** Number of patients per each year of clinic.

Year	No. of Initial Clinic Visits Scheduled	No. of Follow-up Visits
1 July 2010–30 June 2011	14	0
1 July 2011–30 June 2012	44	20
1 July 2012–30 June 2013	45	24
1 July 2013–30 June 2014	52	21
1 July 2014–30 June 2015	76	35
1 July 2015–30 June 2016	84	64

315 total referrals/visits since starting clinic. Five patients had 3 visits since the start of clinic. There were 152 single visits. Four patients did not show up for the initial visit.

**Table 2:** Referrals to a GOLDILOKs^®^ Clinic.

Provider who initiated referral	**Primary Care Provider:** 57 (18.1%)
	Internal providers: 9 (16%)
	External providers: 48 (84%)
	**Subspecialist:** 257 (81.6%)
	Internal providers: 223 (87%)
	External providers: 34 (13%)
	**Self:** 1 (0.3%)
Subspecialist area of practice	Psychiatry/psychology: 188
	Neurology: 18
	Gastroenterology: 16
	Pulmonology/sleep medicine: 8
	Dermatology: 5
	Endocrinology: 4
	Genetics: 4
	Beacon clinic: 3
	Rheumatology: 2
	Allergy/immunology: 2
	Other: 7

**Table 3:** Other reasons for GOLDILOKs^®^ referral.

1. Help transitioning into adult care;
2. To help determine appropriate medication for anxiety and depression;
3. Use of octreotide to assist in control of weight gain;
4. Increased phenobarbital levels, concern for altered phenobarbital metabolism;
5. Evaluation of chronic inflammatory process and medication responses;
6. Evaluation of recurrent hypothermia and medications that may help with thermoregulation;
7. Patient’s family contacted clinical pharmacology to discuss the safety of the prolonged use of sedatives and paralytics because we have assisted with tapering his sedatives during previous hospitalizations. Concerns due to multiple planned future surgeries;
8. Poor tolerance of Oral Iron products. Need recommendations for intravenous dose for Restless Leg Syndrome in pediatric patient;
9. Evaluation of medications to avoid Opsoclonus-Myoclonus Syndrome (OMS).
